# A Novel Rank Aggregation-Based Hybrid Multifilter Wrapper Feature Selection Method in Software Defect Prediction

**DOI:** 10.1155/2021/5069016

**Published:** 2021-11-24

**Authors:** Abdullateef O. Balogun, Shuib Basri, Saipunidzam Mahamad, Luiz Fernando Capretz, Abdullahi Abubakar Imam, Malek A. Almomani, Victor E. Adeyemo, Ganesh Kumar

**Affiliations:** ^1^Department of Computer and Information Science, Universiti Teknologi PETRONAS, Bandar Seri Iskandar 32610, Perak, Malaysia; ^2^Department of Computer Science, University of Ilorin, Ilorin 1515, Nigeria; ^3^Department of Electrical and Computer Engineering, Western University, London, ON, Canada N6A 5B9; ^4^Department of Software Engineering, The World Islamic Sciences and Education University, Amman 11947, Jordan; ^5^School of Built Environment, Engineering and Computing, Leeds Beckett University, Headingley Campus, Leeds LS6 3QS, UK

## Abstract

The high dimensionality of software metric features has long been noted as a data quality problem that affects the performance of software defect prediction (SDP) models. This drawback makes it necessary to apply feature selection (FS) algorithm(s) in SDP processes. FS approaches can be categorized into three types, namely, filter FS (FFS), wrapper FS (WFS), and hybrid FS (HFS). HFS has been established as superior because it combines the strength of both FFS and WFS methods. However, selecting the most appropriate FFS (filter rank selection problem) for HFS is a challenge because the performance of FFS methods depends on the choice of datasets and classifiers. In addition, the local optima stagnation and high computational costs of WFS due to large search spaces are inherited by the HFS method. Therefore, as a solution, this study proposes a novel rank aggregation-based hybrid multifilter wrapper feature selection (RAHMFWFS) method for the selection of relevant and irredundant features from software defect datasets. The proposed RAHMFWFS is divided into two stepwise stages. The first stage involves a rank aggregation-based multifilter feature selection (RMFFS) method that addresses the filter rank selection problem by aggregating individual rank lists from multiple filter methods, using a novel rank aggregation method to generate a single, robust, and non-disjoint rank list. In the second stage, the aggregated ranked features are further preprocessed by an enhanced wrapper feature selection (EWFS) method based on a dynamic reranking strategy that is used to guide the feature subset selection process of the HFS method. This, in turn, reduces the number of evaluation cycles while amplifying or maintaining its prediction performance. The feasibility of the proposed RAHMFWFS was demonstrated on benchmarked software defect datasets with Naïve Bayes and Decision Tree classifiers, based on accuracy, the area under the curve (AUC), and F-measure values. The experimental results showed the effectiveness of RAHMFWFS in addressing filter rank selection and local optima stagnation problems in HFS, as well as the ability to select optimal features from SDP datasets while maintaining or enhancing the performance of SDP models. To conclude, the proposed RAHMFWFS achieved good performance by improving the prediction performances of SDP models across the selected datasets, compared to existing state-of-the-arts HFS methods.

## 1. Introduction

The software development lifecycle (SDLC) is a formal framework that has been specifically planned and built for the production or development of high-quality software systems. To ensure a timely and reliable software system, gradual steps in the SDLC, such as requirement elicitation, software system review, software system design, and software system maintenance, must be closely followed and applied [1–3]. Nevertheless, since the SDLC step-by-step operations are done by professionals, human errors or failures are inevitable. Because of the large scale and dependencies in modules or parts of software systems today, these errors are common and recurring. As a result, if not corrected immediately, these errors will result in unreliable computing structures and, eventually, software failure. That is, the occurrence of errors in software system modules or components will result in flawed and low-quality software systems. Furthermore, flaws in software systems can irritate end-users and customers when the broken software system does not work as intended after the end-user has already wasted limited resources (time and effort) [4–6]. Therefore, it is critical to consider early prediction and discovery of software flaws before product delivery or during the software development process. Early detection or prediction of defective modules or components in a software system allows those modules or components to be corrected momentarily and available resources to be used optimally [7, 8].

Software defect prediction (SDP) is the use of machine learning (ML) methods to determine the defectivity of modules or components in software. SDP, in particular, is the application of ML methods to software features identified by software metrics to detect faults in software modules or components [9–12]. For SDP, some researchers have suggested and applied both supervised and unsupervised ML approaches [13–18]. Nonetheless, the predictive accuracy of SDP models is entirely dependent on the consistency and inherent characteristics of the software datasets used to create them. The magnitude and complexities of software systems are closely related to the software metrics used to characterize the consistency and performance of software systems. That is, large and scalable software systems necessitate several software metric structures to deliver functionality that best reflects the output of those software systems [19–21]. In general, software systems with a large number of features as a result of the accumulation of software metrics are often composed of redundant and irrelevant features, which can be described as a high dimensionality problem. According to research, the high dimensionality problem has a negative impact on the prediction accuracy of SDP models [22, 47]. Researchers agree that the feature selection (FS) approach is an effective method for addressing high-dimensionality problems. For each SDP process, these FS methods essentially selects valuable and critical software features from the initial software defect dataset [23–26].

The application of FS methods results in the creation of a subset of features containing germane and critical features from a collection of trivial and unnecessary features, thus resolving the high dimensionality of the dataset. In other words, FS methods choose the most significant features while retaining dataset performance [27–29]. There are three types of FS methods, namely, filter FS (FFS), wrapper FS (WFS), and hybrid FS (HFS). The FFS method has lower computational complexity but the predictive performance of classification algorithms on such filtered data cannot be guaranteed [30–32]. On the other hand, WFS methods guarantee good predictive performance but come with the cost of high computational complexity and lack of generalizability [31, 33]. The HFS approach combines the strength of both FFS and WFS methods [34, 35]. However, filter rank selection problem and complex search strategies are inherent limitations/drawbacks of HFS methods. In particular, selecting the most appropriate filter method for HFS is difficult, as the performance of FFS methods depends on the choice of datasets and classifiers [36–41]. Also, the local optima stagnation and high computational costs of WFS as a result of large search spaces are inherited by the HFS method [42–44]. Therefore, this research has developed a novel rank aggregation-based hybrid multifilter wrapper feature selection (RAHMFWFS) method for the selection of relevant and irredundant features from software defect datasets. The proposed RAHMFWFS is divided into two stepwise stages. The first stage involves a rank aggregation-based multifilter feature selection (RMFFS) method. RMFFS addresses the filter rank selection problem by aggregating individual rank lists from multiple filter methods and using a rank aggregation method to generate a single, robust, and non-disjoint rank list. In the second stage, the aggregated ranked features are further preprocessed by an enhanced wrapper feature selection (EWFS) method based on a reranking strategy. A dynamic reranking strategy is used to guide the feature subset selection process of the WFS method which in turn reduces the number of wrapper evaluation cycles while maintaining or amplifying its prediction performance. The reranked feature list is then outputted as the optimal feature subset by the proposed RAHMFWFS. The feasibility of the proposed RAHMFWFS was demonstrated on benchmarked software defect datasets with Naïve Bayes and Decision Tree classifiers based on accuracy, area under the curve (AUC), and F-measure values. The proposed RAHMFWFS takes advantage of filter-filter and filter-wrapper relationships to give optimal feature subsets with high predictive performance and also to improve the search strategy in the wrapper in order reduce its evaluation cycle and subsequently improve performance of SDP models.

The main contributions of this study are as follows:

1. To develop a novel rank aggregation-based hybrid multifilter wrapper feature selection (RAHMFWFS) method for the selection of relevant and irredundant features from software defect datasets.
2. To empirically evaluate and validate the performance of RAHMFWFS against rank aggregation-based multifilter feature selection (RMFFS) and enhanced wrapper feature selection (EWFS) methods that are constituents of the proposed RAHMFWFS.
3. To empirically evaluate and validate the performance of RAHMFWFS against existing hybrid FS methods.

The remainder of this paper is structured as follows. Reviews on existing related works are presented in Section 2. Details on proposed RAHMFWFS and experimental methods are described in Section 3. Experimental results are analyzed and discussed in Section 4 and the research is concluded with highlights of future works in Section 5.

## 2. Related Works

High dimensionality is a data quality problem that affects the predictive capabilities of SDP models. In other words, the frequency of redundant and noisy software features as a result of the number and increase in software parameters used to determine the output of a software system has a negative impact on SDP prediction models. Existing research has shown that FS methods can be used to solve the high dimensionality problem. As a result, numerous studies have suggested various FS approaches and investigated their implications on the predictive efficiency of SDP models.

Cynthia et al. [45] evaluated the influence of FS approaches on SDP prediction models. The effect of five FS methods on selected classifiers was specifically investigated. Based on their findings, they concluded that FS methods have a substantial (positive) impact on the prediction output of the chosen classifiers. Nevertheless, the scope of their research (number of FS methods and datasets chosen) was small. Akintola et al. [2] also compared filter-based FS approaches on heterogeneous prediction models, focusing on the following classifiers: principal component analysis (PCA), correlation-based feature selection (CFS), and filtered subset evaluation (FSE). They also discovered that using FS methods in SDP is advantageous because it increases the prediction accuracy of chosen classifiers.

In their research, Balogun et al. [23] explored the effect of FS methods on models in SDP based on applied search methods. The output of eighteen FS methods was evaluated using four classifiers. Their results support the use of FS methods in SDP; however, the impact of FS methods on SDP differs across datasets and classifiers. They reported that filter-based feature selection methods had higher accuracy values than other FS methods tested. Nonetheless, the issue of filter rank selection problem persists because the output of filter-based FS methods is dependent on the dataset and classifier used in the SDP phase. In a similar study, Balogun et al. [24] performed an exhaustive analytical study on the effect of FS approaches on SDP models, focusing on particular discrepancies and anomalies in previous research outlined by Ghotra et al. [46] and Xu et al. [40]. They concluded from their experimental findings that the effectiveness of FS approaches is dependent on the dataset and classifier used. As a result, there are no best FS approaches. Since each filter-based FS approach functions differently, this adds to the support for FFS methods and points to the existence of a filter rank selection problem in SDP.

Wahono et al. [47] improved an ensemble-based SDP model using a metaheuristic-based WFS approach. As a search method for the WFS, they combined Particle Swarm Optimization (PSO) and the genetic algorithm (GA). Their findings demonstrated that the WFS approach improves the ensemble method's predictive efficiency. They then combined PSO and GA as search methods for the proposed WFS method. Their results indicated that the use of WFS method increases the ensemble method's predictive performance. This demonstrates that metaheuristic search methods can be just as effective as traditional Best-First Search (BFS) and Greedy Stepwise Search (GSS) methods. Likewise, in their analysis, Song et al. [48] used two WFS approaches: forward selection and backward elimination. Based on their experimental results, they hypothesized that both forms of WFS benefited SDP models and contended that there is no discernible difference between their performances. However, their emphasis on WFS was restricted to forward selection and backward elimination only. However, metaheuristics and other search methods can be as effective as, if not more effective than, forward selection and backward elimination in WFS methods.

Muthukumaran et al. [49] used 10 FS methods to conduct a systematic analytical analysis on 16 defective datasets (7 FFS, 2 WFS, and 1 embedded method). WFS based on GSS method outperformed other FS methods in their study. The effect of FS methods on SDP models was studied by Rodríguez et al. [50]. Correlation-based FS (CFS), consistency-based FS (CNS), fast correlation-based filter (FCBF), and WFS were empirically contrasted. They stated that datasets with fewer features maintain or outperform the original dataset and that the WFS method outperforms the other FFS approaches that were tested. However, it should be noted that WFS methods are computationally expensive, which may be attributed to the use of standard exhaustive search methods.

Jia [51] has suggested an HFS approach for SDP that combines the strengths of three FFS methods: chi-squared (CS), information gain (IG), and association filter (AF). In that study, the Top *K* features were chosen based on the average rating of each element in the respective rating list. Their findings revealed that models based on the HFS approach outperformed models based on individual FFS methods (CS, IG, AF). Nonetheless, the distorted rankings of each feature will have an impact on the efficacy of averaging rank lists [52]. Furthermore, picking random Top *K* features may not be the right method, since valuable features may be overlooked during the selection process [45].

In another context, Onan [53] deployed a reranking search algorithm with an CNS method for selecting relevant features and reducing the computational complexity of the subset evaluation in the classification of breast cancer. Also, a fuzzy-rough instance selection method was incorporated into the proposed method for instance selection. Experimental findings from the study showed that the proposed HFS method can select relevant features and instances set for model construction. In another related study, Onan and Korukoğlu [54] constructed an ensemble of FS methods for text sentiment classification. They aggregated individual feature lists from diverse FS methods using the GA method. Findings from their results indicated that the proposed ensemble approach can generate more robust and relevant features than respective individual FS methods.

As a result, FS approaches are effective at decreasing or eliminating dataset features and amplifying the performance of models in SDP. Even so, choosing a suitable FFS approach remains a challenge. Also, trapping in local maxima and the high computational cost of WFS methods is an open problem for HFS. Hence, this study proposes a novel rank aggregation-based hybrid multifilter wrapper feature selection (RAHMFWFS) method for the selection of relevant and irredundant features from software defect datasets.

## 3. Methodology

This section contains information on selected classifiers, baseline FFS methods, the proposed RAHMFWFS method, the experimental procedure, datasets studied, and the performance evaluation measures.

### 3.1. Classification Algorithms

Decision Tree (DT) and Naïve Bayes (NB) algorithms were used as prediction models in this analysis due to their high prediction efficiency and their potential for operating on imbalanced datasets [23, 55]. Furthermore, parameter tuning often has little effect on DT and NB. Finally, DT and NB have been used repeatedly in existing SDP studies.  Table 1 contains information on the DT and NB classifiers.

### 3.2. Filter Feature Selection (FFS) Methods

In terms of baseline FFS methods, three FFS methods with varying computational characteristics were chosen for this study. Specifically, chi-square (CS), Relief (REF), and Information Gain (IG) are utilized as baseline FFS methods in this study. CS is a statistic-based FS method that evaluates an attribute's independence from the class label. As an instance-based FS process, REF samples features from a given dataset, compares each sampled feature to its respective neighbourhood, and then assigns a significance score to each feature. IG selects features using an entropy mechanism that is focused on selecting appropriate features by eliminating uncertainties associated with defining the class label when the feature's value is unknown. The broad choice of these FS approaches (CS, REF, and IG) is based on the actual research findings [23, 24]. More information on the chosen FS methods can be found in [56–60].

### 3.3. The Rank Aggregation-Based Hybrid Multifilter Wrapper Feature Selection (RAHMFWFS) Method

**Figure 1:**
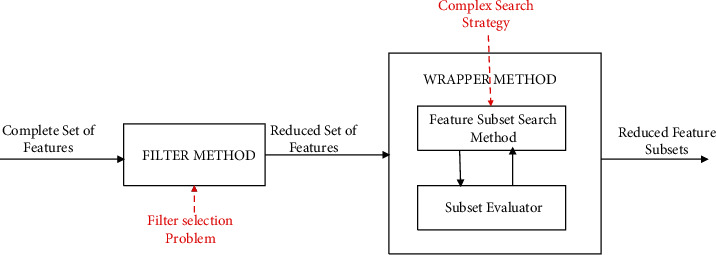
Problems with conventional HFS methods.

Finding a way to hybridize FFS and WFS methods to maintain high performance and a generalizable result is still an open research issue. This is due to the filter rank selection problem and the complex search method used in the FFS and WFS parts of HFS methods, respectively. That is, HFS methods inherit both the best features and some of the problems of FFS and WFS, as depicted in Figure 1.

**Figure 2:**
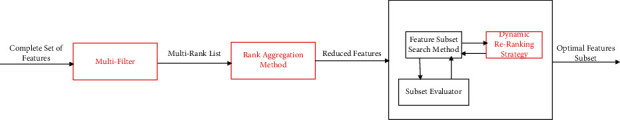
Proposed solutions to problems in conventional HFS methods.

However, the filter rank selection problem and complex search method in HFS methods can be addressed by introducing multifilter and reranking mechanisms into FFS and WFS parts of the HFS method, respectively.  Figure 2 presents a conceptual framework on proposed solutions to problems in the HFS method.

**Figure 3:**
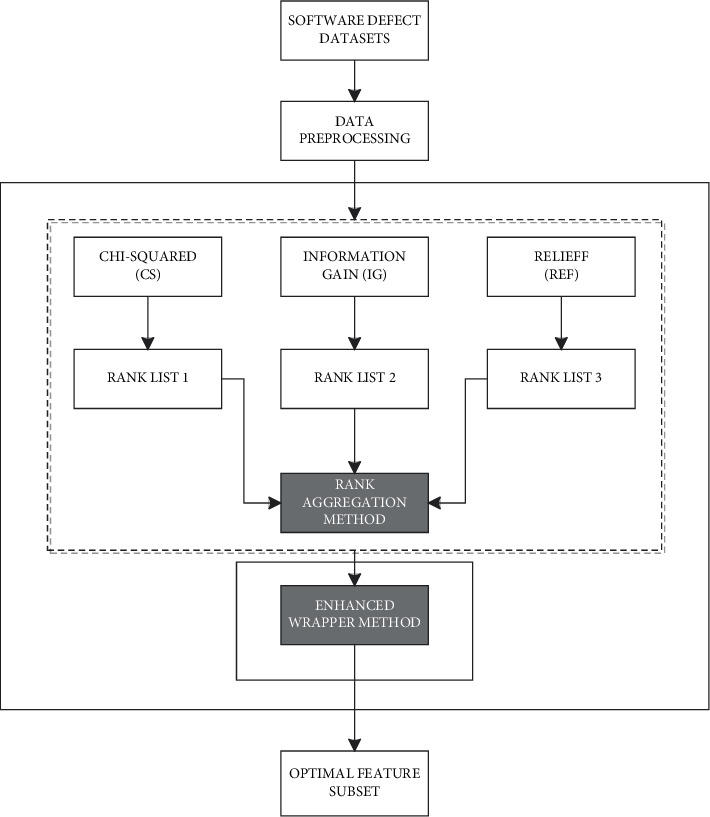
Conceptual framework of RAHMFWFS method.

Consequently, this study proposes the RAHMFWFS method, which takes filter-filter (multifilter methods) and filter-wrapper (HFS methods) relationships into account to provide optimal feature subsets with high predictive performance. The conceptual framework of the proposed RAHMFWFS is presented in Figure 3.

The proposed RAHMFWFS method aims to take into account and incorporate the computational capabilities of multiple independent FFS and enhanced WFS methods. RAHMFWFS addresses high dimensionality issues in SDP tasks by selecting optimal features using multiple FFS methods and reranking strategy-based WFS methods.

Specifically, the proposed RAHMFWFS can be divided into two sequential stages: the rank aggregation-based multifilter FS (RMFFS) stage and the enhanced wrapper FS (EWFS) stage.

#### 3.3.1. Rank Aggregation-Based Multifilter FS (RMFFS) Stage

As depicted in Figure 2, RMFFS is the first stage of the proposed RAHMFWFS and it primarily aims to resolve the filter rank selection problem that is inherent in HFS methods. Multiple rank lists are generated by the independent FFS methods (CS, IG, and REF). These selected FFS methods are mutually exclusive with distinct underlying computational characteristics and are chosen based on findings from existing studies [23, 24]. The multiple rank lists that are developed are then aggregated using the rank aggregation functions shown in Table 2. Using the significance score assigned to each attribute on the individual rank lists, each rank aggregation function combines the various rank lists into a single aggregated rank list.

In particular, the *minimum* (*min*) and *maximum* (*max*) rank aggregation functions select features using the minimum and maximum significance score provided by the generated aggregated rank list, respectively, while the *arithmetic mean* (*mean*) rank aggregation function aggregates multiple rank lists into a single aggregated rank list by calculating the arithmetic mean of the significance scores assigned to each element on the individual rank lists. The resulting rank lists generated by the respective rank aggregation functions (*min, max, and mean*) are ensembled using the majority voting mechanism to produce a single aggregated rank list. As observed, the rank lists generated from each of the rank aggregators are mutually exclusive subsets of features that are considered relevant and important based on each of the rank aggregator methods.

In the end, a backtracking function is applied to the aggregated rank list to validate the significance of each feature in the list by assessing the significance score of features on the aggregated rank list with corresponding significance scores of feature in the baseline FFS methods (in this case CS, IG, and REF). The backtracking function aims to further refine the features on the generated aggregated rank list in line with the respective significance score of features from the FFS methods. This is a logical procedure to further remove irrelevant or redundant features from the generated aggregated rank list.

#### 3.3.2. Enhanced Wrapper Feature Selection (EWFS) Stage

EWFS is the second stage of the proposed RAHMFWMFS method that further refines the HFS process by deploying a reranking mechanism that dynamically and iteratively selects features. That is, the EWFS method incrementally selects features while considering previously selected features in its search space. In the beginning, an entropy measure is used to rank features and then the ranked features are passed through an incremental wrapper method. However, it is only the first *B* ranked features selected by the entropy by log_2_  *N* that are passed to the incremental wrapper method. Thereafter, the remaining features in *B* are reranked using the conditional mutual information maximization (CMIM) algorithm while considering the initially selected features. Using a multiobjective cuckoo search method, CMIM balances and selects features by optimizing their shared knowledge with the class label while minimizing any codependency that might occur between or within features. The incremental wrapper approach is then deployed on the newly ranked set, which has been initialized by the features first selected from *B*. This procedure is replicated until no changes are detected in the chosen features. Consequently, the EWFS method reduces the number of wrapper evaluations, since only a few features will be considered during each iteration, thus retaining or improving the prediction efficiency of the chosen features.

In summary, the proposed RAHMFWMFS is developed to take advantage of filter-filter and filter-wrapper relationships, select optimal feature subsets with high predictive performance, and also improve the search strategy in the wrapper to reduce its evaluation cycle and subsequently improve the performance of SDP models. The pseudocode for the proposed RAHMFWFS method is presented in Algorithm 1.

### 3.4. Software Defect Datasets

This study's experimentation used defect datasets from four publicly accessible repositories. Twenty-five datasets of differing granularities were chosen from the PROMISE, NASA, AEEEM, and ReLink repositories. Shepperd et al.'s [61] variant of defect datasets from the NASA repository is included in this analysis. The datasets are made up of software features created by static code metrics. The size and complexity of the source code were used to calculate static code metrics [25, 47]. The PROMISE repository houses defect datasets extracted from object-oriented metrics as well as external data from app modules. This additional data comes from the apache software [22, 25, 62]. The datasets in the ReLink repository are extracted from source code information from version control. Wu et al. [63] developed these datasets as linkage evidence, and they have been widely used in previous SDP studies [64–66]. Finally, the AEEEM datasets have program features derived from source code metrics such as shift metrics, entropy, and source code churn [25, 47, 62, 67].  Table 3 contains a list of these datasets.

### 3.5. Performance Evaluation Metrics

In terms of efficiency, SDP models based on the proposed method and other methods were evaluated using accuracy, the area under the curve (AUC), and F-measure values. These metrics are widely used in SDP studies to evaluate the efficiency of SDP models [8, 68].

(i) Accuracy is the sum or percentage of data that is correctly calculated out of the total amount of data, as seen in the following equation:
  (1) $$\begin{array}{c} \text{Accuracy}=\frac{\text{TP}+\text{TN}}{\text{TP}+\text{FP}+\text{FN}+\text{TN}}x\;100\%, \end{array}$$
(ii) The F-measure is calculated using the harmonic mean of the precision and recall values of the observed data. The formula for calculating the F-measure value is as follows:

(2) $$\begin{array}{c} \text{F}-\text{Measure}=2\times \left(\frac{\text{Precision}\times \text{Recall}}{\text{Precision}+\text{Recall}}\right). \end{array}$$

The area under curve (AUC) is a measure of the trade-off between true and false positives. It indicates an overall performance evaluation across all classification thresholds.

Recall=(TP/TP+FN), Precision=(TP/TP+FP), TP = True Positive (represents accurate prediction), FP = False Positive (represents inaccurate prediction), TN = True Negative (represents accurate misprediction), and FN = False Negative (represents inaccurate misprediction).

### 3.6. Experimental Framework

**Figure 4:**
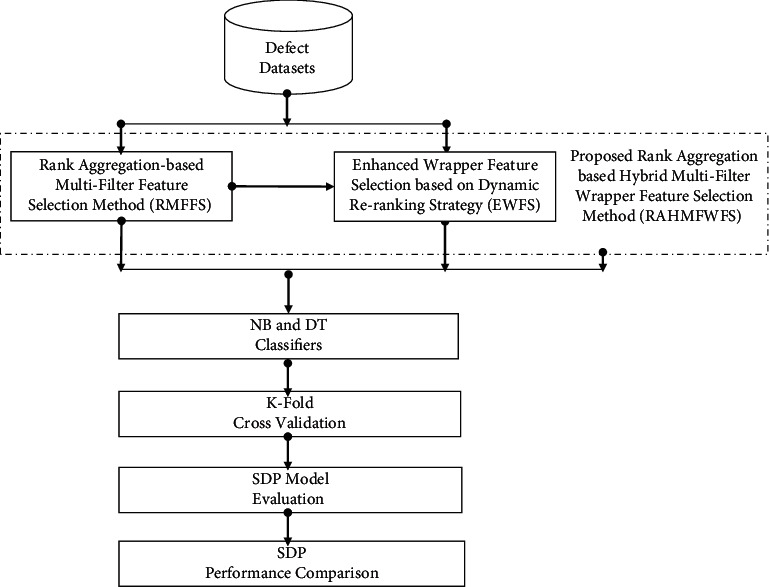
Experimental framework.

This section presents and discusses the experimental design of this study, as illustrated in Figure 4.

To evaluate the effects of the proposed RAHMFWFS method on SDP model prediction results, software defect datasets as presented in Table 3 were used to develop SDP models based on NB and DT classifiers (see Table 1). Multiple experiments and analyses were conducted to obtain an impartial and standard performance comparison of the resulting SDP models. Firstly, the performance of the proposed RAHMFWFS method was compared with those of existing hybrid FS methods. Specifically, RAHMFWFS was compared with IWSS [69] and IWSSr [70]. Secondly, the proposed RAHMFWFS method was compared with RMFFS and EWFS. All experiments were conducted on the software defect datasets that were studied (see Table 3) with NB and DT classifiers based on average accuracy value, average AUC value, and average F-measure value. The Scott-KnottESD statistical rank test was carried out on the results to determine the significant difference in their respective average performance metric values. The essence of these experiments and statistical analysis is to evaluate and validate the performance of the RAHMFWFS method against existing HFS methods (IWSS and IWSSr) and its constituent (RMFFS and EWFS) methods.

Results and observations based on the above-mentioned experiments were used to answer the following research questions:

  RQ1: How effective is the proposed RAHMFWFS method compared to existing HFS (IWSS and IWSSr) methods?
  RQ2: How effective is the proposed RAHMFWFS method compared to its constituent (RMFFS and EWFS) methods?

SDP models based on the experiments mentioned above were trained and evaluated using the 10-fold cross-validation (CV) technique. The CV technique can guard against data heterogeneity problems that can arise in defective datasets. Furthermore, the CV technique is known to generate models with low bias and variance [71–75]. Experimentation was carried out using the Weka machine learning library [76], *R* lang. [77], and Origin Plot.

## 4. Results and Discussion

This section presents and discusses the performance of the proposed rank aggregation-based hybrid multifilter wrapper feature selection method (RAHMFWFS). Also, the performance of the proposed RAHMFWFS is compared with those of existing hybrid FS methods and the duo of RMFFS and EWFS.

Table 4 presents the experimental results of the proposed RAHMFWFS method with NB and DT classifiers on 25 defect datasets based on accuracy, AUC, and F-measure values. From Table 4, it can be observed that RAHMFWFS with NB and DT classifiers had an average accuracy value of 82.67% and 83.80%, respectively. This shows that RAHMFWFS with NB and DT classifiers correctly predicts defects in SDP, which translates to a good prediction performance of models based on RAHMFWFS. In addition, RAHMFWFS with NB and DT classifiers recorded average AUC values of 0.802 and 0.732, respectively, and average F-measure values of 0.823 and 0.84, respectively. The average AUC values of RAHMFWFS on NB and DT are above average (0.5), which means that the prediction is not subject to chance. The high average AUC values of RMFFS on NB (0.802) and DT (0.732) further support its high accuracy value such that the developed models have a high chance of distinguishing between defective and nondefective modules or components. Furthermore, high average F-measure values of RAHMFWFS on NB (0.823) and DT (0.840) show that the developed models have good precision and recall. Thus, the high F-measure RAHMFWFS with NB and DT indicates that the developed models are precise and robust in identifying defective modules or components.

Based on the preceding observations, the positive prediction performance of NB and DT models based on the RAHMFWFS method demonstrates its capability to address the filter rank selection problem, local optima stagnation, and subsequent high dimensionality problems by exploiting the filter-filter relationship by aggregating rank lists from multiple FFS methods into a single robust rank list and reducing the wrapper evaluation cycle of its wrapper component while maintaining or enhancing the prediction performance of SDP models.

The performance comparison of RAHMFWFS against existing IWSS and IWSSr methods is presented and discussed in the next subsection.

### 4.1. Performance Comparison of RAHMFWFS and Existing HFS (IWSS and IWSSr) Methods

In this subsection, the performance of the proposed RAHMFWFS is compared with those of state-of-the-art HFS. In particular, the prediction performances of NB and DT models based on the proposed RAHMFWFS are compared and contrasted with hybrid Incremental Wrapper-based Subset Selection (IWSS) as proposed by [69] and hybrid Incremental Wrapper-based Subset Selection with replacement and early stopping (IWSSr) as proposed by [70].

**Figure 5:**
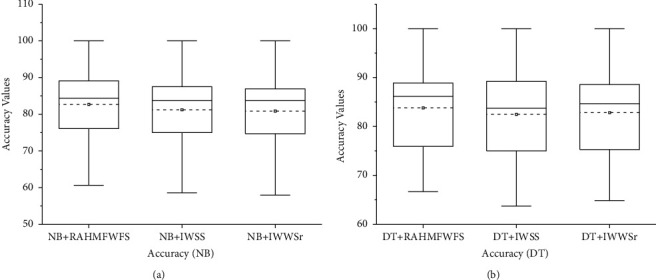
Box-plot representation of accuracy values of NB and DT models based on RAHMFWFS and existing HFS methods.

Figure 5 presents the box-plot representation of average accuracy values of NB and DT models with RAHMFWFS and existing HFS methods (IWSS and IWSSr). In terms of average accuracy values, the proposed RAHMFWFS with NB and DT classifiers recorded superior average accuracy values when compared with NB and DT models based on IWSS and IWSSr. Specifically, RAHMFWFS with NB and DT recorded average accuracy values of 82.67% and 83.8%, respectively, compared with IWSS (NB: 81.21%, DT: 82.47%) and IWSSr (NB: 80.87%, DT: 82.82%). Thus, NB and DT models based on EWFS outperformed models based on IWSS by +1.8% and +1.6% and IWSSr by +2.23% and +1.18%, based on average accuracy values. These analyses demonstrate the superiority of RAHMFWFS over IWSS and IWSSr methods based on average accuracy values.

**Figure 6:**
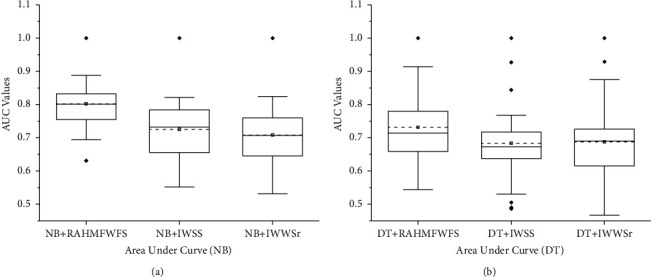
Box-plot representation of AUC values of NB and DT models based on RAHMFWFS and existing HFS methods.

Additionally, Figure 6 presents the box-plot representation of average AUC values of NB and DT models with RAHMFWFS and existing HFS methods (IWSS and IWSSr). NB and DT classifiers with the RAHMFWFS method recorded higher average AUC values when compared against models based on IWSS and IWSSr methods. RAHMFWFS with NB and DT had average AUC values of 0.802 and 0.732, respectively, compared with IWSS (NB: 0.725, DT: 0.684) and IWSSr (NB: 0.708, DT: 0.688). Specifically, NB and DT models based on RAHMFWFS outperformed models based on IWSS by +10.6% and +7.02% and IWSSr by +13.28% and +6.4%, respectively, based on average AUC values.

**Figure 7:**
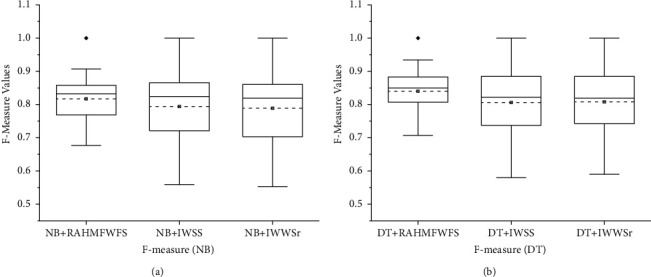
Box-plot representation of F-measure values of NB and DT models based on RAHMFWFS and existing HFS methods.

Furthermore, Figure 7 shows the box-plot representation of average F-measure values of NB and DT models with RAHMFWFS and existing HFS methods (IWSS and IWSSr). Prediction models (NB and DT) with RAHMFWFS methods recorded average F-measure values of 0.823 and 0.84, respectively, which are superior to average F-measure values of models based on IWSS (NB: 0.794, DT: 0.806) and IWSSr (NB: 0.789, DT: 0.808) methods. In particular, NB and DT models based on RAHMFWFS outperformed models based on IWSS by +3.65% and +4.22% and IWSSr by +4.31% and +3.96, respectively, based on average F-measure values. Hence, NB and DT models with RAHMFWFS recorded superior F-measure values when compared against models with existing HFS (IWSS and IWSSr) methods.

**Figure 8:**
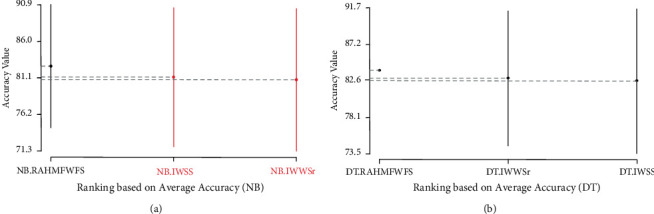
Scott-KnottESD statistical rank test results of RAHMFWFS and existing HFS methods on NB and DT based on average accuracy value. (a) Average accuracy value of NB. (b) Average accuracy value of DT.

Figure 8 shows the Scott-KnottESD statistical rank test results of the proposed RAHMFWFS method and IWSS and IWSSr methods on NB and DT, based on average accuracy values. As shown in Figure 8(a), the average accuracy performances of NB based on the RAHMFWFS method are superior to IWSS and IWSSr as the proposed RAHMFWFS outranks both IWSS and IWSSr. That is, there is a statistically significant difference in the average accuracy value of the NB model based on RAHMFWFS when compared with NB models based on IWSS and IWSSr. Contrary to this observation is the case of the proposed RAHMFWFS with DT models. As shown in Figure 8(b), although RAHMFWFS with DT had superior average accuracy values when compared with DT models based on IWSS and IWSSr, there is no statistically significant difference in their respective average accuracy values. That is, there is not much difference between the average accuracy values of RAHMFWFS and existing HFS (IWSS and IWSSr) methods with the DT classifier.

**Figure 9:**
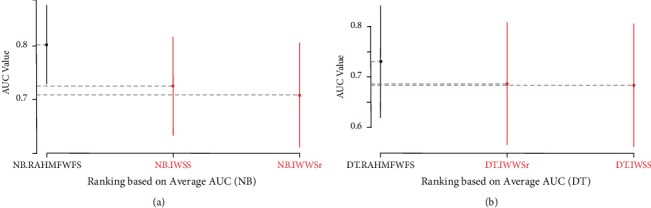
Scott-KnottESD statistical rank test results of RAHMFWFS and existing HFS methods on NB and DT based on average AUC value. (a) Average AUC value of NB. (b) Average AUC value of DT.

Also, Figure 9 presents the Scott-KnottESD statistical rank test results of the proposed RAHMFWFS and IWSS and IWSSr methods on NB and DT based on average AUC values. There are significant statistical differences in the average AUC values of NB and DT models based on RAHMFWFS when compared to IWSS and IWSSr. Specifically, NB and DT models based on RAHMFWFS outrank and outperform the existing IWSS and IWSSr methods. The high accuracy and AUC values of NB and DT models based on RAHMFWFS thus demonstrate that RAHMFWFS can avoid overfitting of models, in contrast to IWSS and IWSSr.

**Figure 10:**
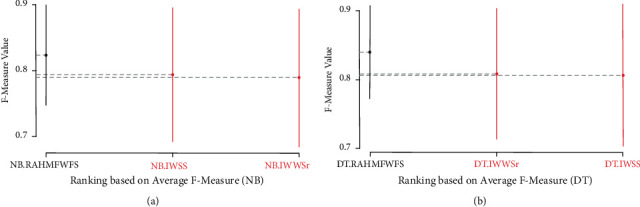
Scott-KnottESD statistical rank test results of RAHMFWFS and existing HFS methods on NB and DT based on average accuracy value. (a) Average F-measure value of NB. (b) Average F-measure value of DT.

Furthermore, similar findings are observed in the case of average F-measure value as presented in Figure 10. NB and DT models based on the RAHMFWFS method outranked and outperformed IWSS and IWSSr methods, since there were significant statistical differences in the average F-measure values in favour of NB and DT models based on RAHMFWFS.

Table 5 tabulates and summarizes the Scott-KnottESD statistical rank test results of NB and DT models based on RAHMFWFS and existing HFS (IWSS and IWSSr) methods. As presented in Table 5, it can be observed that NB and DT models based on RAHMFWFS are superior and rank best when compared against NB and DT models based on IWSS and IWSSr methods on average accuracy, average AUC, and average F-measure values. Thus, these observations indicate the superiority of RAHMFWFS over existing hybrid IWSS and IWSSr methods in selecting relevant features in SDP processes.

### 4.2. Performance Comparison of RAHMFWFS against RMFFS and EWFS Methods

In this subsection, the performance of the proposed RAHMFWFS is compared against those of its constituent methods, RMFFS and EWFS, respectively. This comparative analysis of performance is conducted to further ascertain and establish the effectiveness and efficacy of the proposed RAHMFWFS method for the feature selection task in SDP processes.

**Figure 11:**
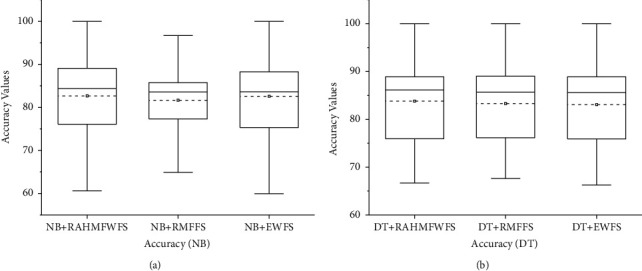
Box-plot representation of accuracy values of NB and DT models based on RAHMFWFS, RMFFS, and EFWS methods.

The box-plot representation of average accuracy values of NB and DT models with RAHMFWFS against RMFFS and EWFS methods is presented in Figure 11. Based on average accuracy values, NB and DT models with proposed RAHMFWFS recorded superior average accuracy values when compared with NB and DT models based on RMFFS and EWFS methods. In particular, RAHMFWFS with NB and DT recorded average accuracy values of 82.67% and 83.8%, respectively, compared with IWSS (NB: 81.21%, DT: 82.47%) and IWSSr (NB: 81.67%, DT: 83.31%). Although the difference in the increased average accuracy values of NB and DT models based on RAHMFWFS against RMFFS and EWFS was small, RAHMFWFS still outperformed both RMFFS (NB: +1.22% and DT: +0.59%) and EWFS (NB: +0.12% and DT: +0.88%) based on average accuracy values. This observation indicates the superiority of RAHMFWFS over RMFFS and EWFS methods based on average accuracy values.

**Figure 12:**
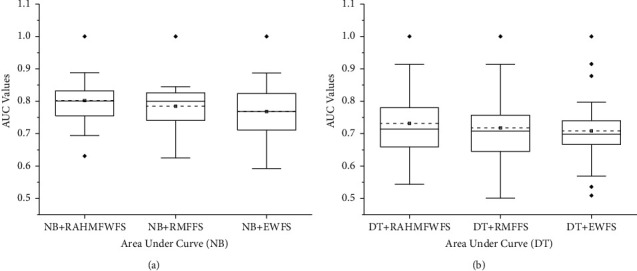
Box-plot representation of AUC values of NB and DT models based on RAHMFWFS, RMFFS, and EFWS methods.

Additionally, Figure 12 presents the box-plot representation of average AUC values of NB and DT models with RAHMFWFS, RMFFS, and EWFS methods. It can be observed that models based on NB and DT classifiers with the RAHMFWFS method recorded higher average AUC values when compared against models based on RMFFS and EWFS methods. RAHMFWFS with NB and DT had average AUC values of 0.802 and 0.732, respectively, compared with RMFFS (NB: 0.784, DT: 0.718) and EWFS (NB: 0.768, DT: 0.708). In particular, NB and DT models based on RAHMFWFS outperformed models based on RMFFS by +2.3% and +1.95% and EWFS by +4.43% and +3.39%, respectively, based on average AUC values.

**Figure 13:**
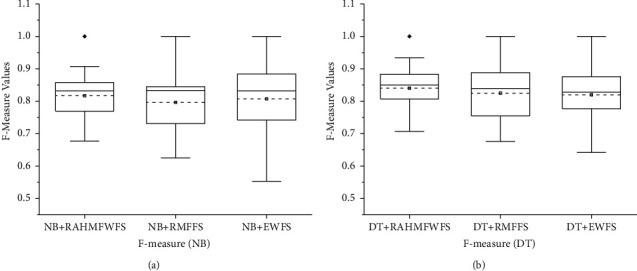
Box-plot representation of F-measure values of NB and DT models based on RAHMFWFS, RMFFS, and EFWS methods.

Concerning F-measure values, Figure 13 showcases the box-plot representation of average F-measure values of NB and DT models with RAHMFWFS, RMFFS, and EWFS methods. Prediction models (NB and DT) with RAHMFWFS methods recorded average F-measure values of 0.823 and 0.84, respectively, outperforming models based on RMFFS (NB: 0.797, DT: 0.825) and EWFS (NB: 0.821, DT: 0.826) methods. That is, NB and DT models based on RAHMFWFS were superior to models based on RMFFS by +3.26% and +1.81% and EWFS by +0.24% and +1.69, respectively, based on average F-measure values. Based on the preceding F-measure results, models with RAHMFWFS recorded superior F-measure values and outperformed models with proposed RMFFS and EWFS methods.

Based on the experimental results, RAHMFWFS, which is a hybrid of RMFFS and EWFS methods, was superior to the individual RMFFS and EWFS methods. This observation can be explained by the further enhancement of the performance of RMFFS by the EWFS as designed in the proposed RAHMFWFS.

**Figure 14:**
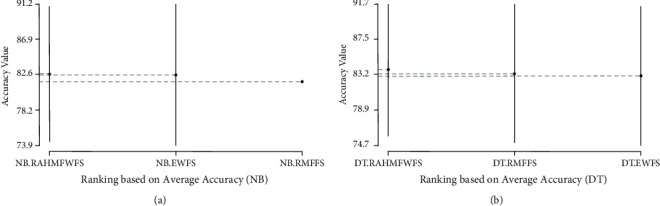
Scott-KnottESD statistical rank test results of RAHMFWFS, RMFFS, and EWFS methods on NB and DT based on average accuracy value. (a) Average accuracy value of NB. (b) Average accuracy value of DT.

**Figure 15:**
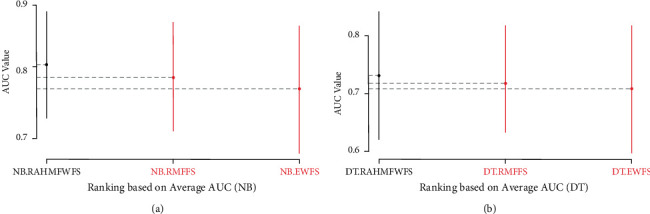
Scott-KnottESD statistical rank test results of RAHMFWFS, RMFFS, and EWFS methods on NB and DT based on average AUC value. (a) Average AUC value of NB. (b) Average AUC Value of DT.

**Figure 16:**
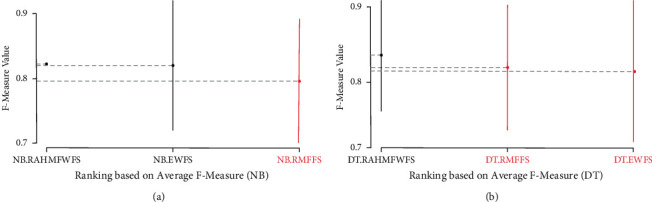
Scott-KnottESD statistical rank test results of RAHMFWFS, RMFFS, and EWFS methods on NB and DT based on average F-measure value. (a) Average F-measure value of NB. (b) Average F-measure value of DT.

Further statistical analyses using Scott-KnottESD were conducted to ascertain the statistical significance in the performance of RAHMFWFS over RMFFS and EWFS methods based on average accuracy, average AUC, and average F-measure values. These are presented in Figures 14–16.

Figure 14 presents the Scott-KnottESD statistical rank test results of RAHMFWFS, RMFFS, and EWFS methods on NB and DT, based on average accuracy values. As depicted in Figure 14(a), it can be observed that average accuracy performances of NB based on RAHMFWFS, RMFFS, and EWFS methods rank at the same position, although the average accuracy values of NB with RAHMFWFS are still somewhat superior. That is, there is no statistically significant difference in the average accuracy values of NB with RAHMFWFS against RMFFS and EWFS methods. A similar observation can be seen in average accuracy values of the DT model with RAHMFWFS, RMFFS, and EWFS methods. As seen in Figure 14(b), RAHMFWFS with DT had superior average accuracy values when compared with DT models based on RMFFS and EWFS; however, there is no statistically significant difference in their respective average accuracy values. That is, there is not much difference between the average accuracy values of RAHMFWFS, RMFFS, and EWFS methods with the DT classifier.

Figure 15 presents the Scott-KnottESD statistical rank test results of RAHMFWFS, RMFFS, and EWFS methods on NB and DT, based on average AUC values. Significant statistical differences can be observed in the average AUC values of NB and DT models based on RAHMFWFS when compared to RMFFS and EWFS methods, respectively. Specifically, NB and DT models based on RAHMFWFS outrank and outperform the proposed RMFFS and EWFS methods. These findings indicate that the high accuracy and AUC values of NB and DT models based on RAHMFWFS are an indicator that RAHMFWFS is superior to both proposed RMFFS and EWFS methods.

Furthermore, Figure 16 shows the Scott-KnottESD statistical rank test results of RAHMFWFS, RMFFS, and EWFS methods on NB and DT based on average F-measure values. As shown in Figure 16(a), it can be observed that although RAHMFWFS with NB recorded a superior average F-measure value when compared with RMFFS and EWFS, there is no statistically significant difference in their respective average F-measure values. This means that there is not much difference between the average F-measure values of RAHMFWFS, RMFFS, and EWFS methods with NB classifier. In contrast, as shown in Figure 16(b), the performance of DT with the RAHMFWFS method is superior to RMFFS and EWFS as RAHMFWFS outranks both RMFFS and EWFS. This means that there is a statistically significant difference in average F-measure values of the DT model based on RAHMFWFS when compared with NB models based on RMFFS and EWFS methods.

As presented in Table 6, it can be observed that NB and DT models based on RAHMFWFS are superior and rank best in most cases when compared against NB and DT models based on RMFFS and EWFS methods on average accuracy, average AUC, and average F-measure values. Thus, these observations indicate and support the superiority of RAHMFWFS over RMFFS and EWFS methods in selecting relevant features in SDP processes.

In summary, the proposed RAHMFWFS focuses on selecting optimal features for the SDP process while maintaining or enhancing the prediction performance of SDP models. The experimental results have demonstrated the superiority of the proposed RAHMFWFS, as it outperformed the existing HFS methods based on empirical and statistical test results. Also, RAHMFWFS significantly outperformed RMFFS and EWFS in most of the experiments. This can be attributed to the fact that RAHMFWFS is a hybrid of the RMFFS and EWFS methods. Therefore, these results showed that RAHMFWFS can be effectively and practically used for the high dimensionality problem in SDP processes.

To provide answers to the research questions (RQs) raised in this study (see Section 3.6), the following observations were drawn based on the experimental results obtained:

  RQ1: *How effective is the proposed RAHMFWFS method compared to existing hybrid FS (IWSS and IWSSr) methods?*
  The proposed RAHMFWFS method indeed produced a significant improvement as compared with existing hybrid FS methods such as IWSS [69] and IWSSr [70]. Specifically, the prediction performances of NB and DT models based on the RAHMFWFS method, as presented in Section 4.1 of this paper, outperformed those of models based on IWSS and IWSSr methods significantly based on accuracy, AUC, and F-measure values.
  RQ2: *How effective is the proposed RAHMFWFS method compared to its constituent (RMFFS and EWFS) methods?*

Similarly, the proposed RAHMFWFS method outperformed the RMFFS and EWFS methods.

The RAHMFWFS method recorded a superior and significant positive effect on the prediction performances of the NB and DT classifier as compared to RMFFS and EWFS methods. That is, RAHMFWFS not only selects optimal features better than RMFFS and EWFS but also handles filter rank selection and local optima stagnation problems.

## 5. Conclusion

Finding a way to hybridize FFS and WFS methods to maintain high performance and a generalizable result is still an open research issue. Based on this premise, this study has proposed a novel rank aggregation-based hybrid multifilter wrapper feature selection (RAHMFWFS) method for the selection of relevant and irredundant features from software defect datasets. The proposed RAHMFWFS method was developed to take advantage of filter-filter and filter-wrapper relationships to provide optimal feature subsets with high predictive performance and also to improve the search strategy in the wrapper to reduce its evaluation cycle and subsequently improve the performance of SDP models. The experimental findings demonstrated the efficacy of the RAHMFWFS technique, which had a greater positive impact on the prediction performances of NB and DT models compared to existing HFS methods in the majority of cases. That is, the proposed RAHMFWFS method was able to address filter rank selection and local stagnation problems in HFS methods by deploying and integrating a rank aggregation-based multifilter method and a dynamic reranking strategy solution for both problems, respectively. Also, the RAHMFWFS method recorded the ability to select optimal features from SDP datasets while maintaining or enhancing the performance of SDP models.

As a continuation of this study, in-depth analysis and investigation on the number of FFS methods in developing multifilter FS methods will be conducted. The relationship between diversity and the number of FFS methods to be considered for the multifilter FS method is also worth exploring. Finally, the possibility of using informed approaches that can resolve ambiguities that may result from combining rank lists ought to be considered. Perhaps, novel approaches that address redundancy introduced during aggregation of individual rank lists can also be developed.

## Figures and Tables

**Algorithm 1:**
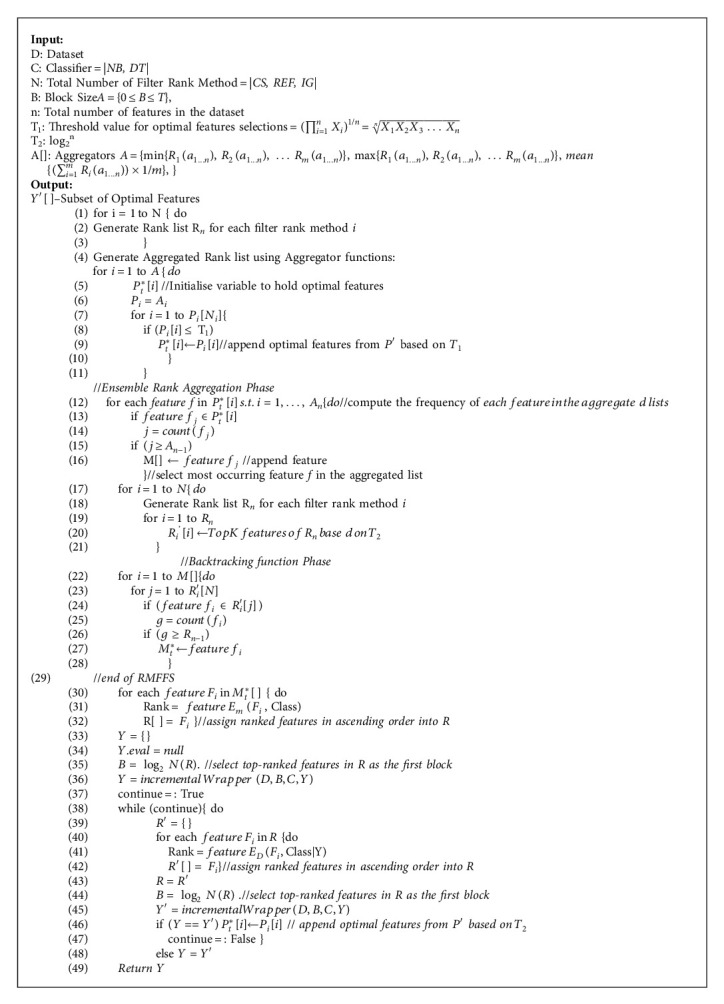
Pseudocode of proposed rank aggregation-based hybrid multifilter wrapper FS (RAHMFWFS) method.

**Table 1:** Selected classifiers.

Classification algorithms	Parameter settings
Decision Tree (DT)	ConfidenceFactor = 0.25; MinObj = 2
Naïve Bayes (NB)	NumDecimalPlaces = 2; NumAttrEval = Normal Dist.

**Table 2:** Rank aggregation methods.

Aggregators	Formula	Description
Min ()	min{*R*_1_(*a*_1…*n*_), *R*_2_(*a*_1…*n*_), … *R*_*m*_(*a*_1…*n*_)}	Selects the *minimum* of the relevance scores produced by the aggregated rank list
Max ()	max{*R*_1_(*a*_1…*n*_), *R*_2_(*a*_1…*n*_), … *R*_*m*_(*a*_1…*n*_)}	Selects the *maximum* of the relevance scores produced by the aggregated rank list
Mean ()	*mean*{(∑_*i*=1_^*m*^*R*_*i*_(*a*_1…*n*_)) × (1/*m*)	Selects the *mean* of the relevance scores produced by the aggregated rank list

**Table 3:** Selected software defect datasets.

Datasets	Number of features	Number of modules
EQ	62	324
JDT	62	997
ML	62	1862
PDE	62	1497
CM1	38	327
KC1	22	1162
KC2	22	522
KC3	40	194
MW1	38	250
PC1	38	679
PC3	38	1077
PC4	38	1287
PC5	39	1711
ANT	22	292
CAMEL	21	339
JEDIT	22	312
REDKITOR	21	176
TOMCAT	22	852
VELOCITY	21	196
XALAN	22	797
SAFE	27	56
ZXING	27	399
APACHE	27	194
ECLIPSE	19	1065
SWT	18	1485

**Table 4:** Experimental results of RAHMFWFS based on accuracy, AUC, and F-measure.

Datasets	Accuracy value (%)		AUC value		F-measure value	
	RAHMFWFS		RAHMFWFS		RAHMFWFS	
	NB	DT	NB	DT	NB	DT
EQ	74.38	75.93	0.818	0.79	0.716	0.761
JDT	84.75	84.25	0.831	0.76	0.838	0.817
ML	85.12	87.11	0.735	0.689	0.842	0.835
PDE	83.23	86.77	0.755	0.655	0.832	0.823
CM1	84.4	85.32	0.744	0.544	0.823	0.807
KC1	76.07	86.16	0.888	0.644	0.718	0.725
KC2	84.67	84.1	0.818	0.78	0.83	0.818
KC3	79.9	83	0.886	0.85	0.769	0.803
MC2	72.8	74.4	0.704	0.777	0.731	0.707
MW1	89.6	91.2	0.782	0.615	0.869	0.89
PC1	91.46	91.9	0.792	0.659	0.907	0.898
PC3	87.5	87.56	0.806	0.884	0.934	0.934
PC4	89.9	88.89	0.845	0.87	0.858	0.874
PC5	74.34	75.34	0.694	0.673	0.691	0.882
SAFE	76.79	69.64	0.866	0.763	0.752	0.85
ZXING	74.16	73.92	0.832	0.561	0.809	0.8
APACHE	74.16	75.65	0.755	0.72	0.718	0.716
ECLIPSE	100	100	1	1	1	1
SWT	83.97	90.16	0.885	0.914	0.835	0.883
ANT	89.38	88.36	0.801	0.71	0.89	0.874
JEDIT	83.01	80.41	0.796	0.714	0.791	0.864
REDKITOR	90.34	89.77	0.774	0.686	0.883	0.885
TOMCAT	89.08	92.14	0.826	0.694	0.892	0.896
VELOCITY	87.24	86.22	0.789	0.741	0.858	0.853
XALAN	60.6	66.7	0.631	0.595	0.788	0.808
Average	82.67	83.80	0.802	0.732	0.823	0.840

**Table 5:** Summary of Scott-KnottESD statistical rank test of RAHMFWFS and existing HFS methods on studied datasets.

Statistical ranking based on average accuracy				Statistical ranking based on average AUC				Statistical ranking based on average F-measure			
NB		DT		NB		DT		NB		DT	
Rank	FS methods	Rank	FS methods	Rank	FS methods	Rank	FS methods	Rank	FS methods	Rank	FS methods
1	RAHMFWFS	1	RAHMFWFS, IWSS, IWSSr	1	RAHMFWFS	1	RAHMFWFS	1	RAHMFWFS	1	RAHMFWFS
2	IWSS, IWSSr			2	IWSS, IWSSr	2	IWSS, IWSSr		IWSS, IWSSr	2	IWSS, IWSSr

**Table 6:** Summary of Scott-KnottESD statistical rank test of RAHMFWFS, RMFFS, and EWFS methods on studied datasets.

Statistical ranking based on average accuracy				Statistical ranking based on average AUC				Statistical ranking based on average F-measure			
NB		DT		NB		DT		NB		DT	
Rank	FS methods	Rank	FS methods	Rank	FS methods	Rank	FS methods	Rank	FS methods	Rank	FS methods
1	RAHMFWFS, RMFFS, EFWS	1	RAHMFWFS, RMFFS, EFWS	1	RAHMFWFS	1	RAHMFWFS	1	RAHMFWFS, EWFS	1	RAHMFWFS
				2	RMFFS, EFWS	2	RMFFS, EFWS		RMFFS	2	RMFFS, EWFS

## Data Availability

Data (experimented datasets and results) are available upon request.
